# Freeze and Thaw of CD4^+^CD25^+^Foxp3^+^ Regulatory T Cells Results in Loss of CD62L Expression and a Reduced Capacity to Protect against Graft-versus-Host Disease

**DOI:** 10.1371/journal.pone.0145763

**Published:** 2015-12-22

**Authors:** Mareike Florek, Dominik Schneidawind, Antonio Pierini, Jeanette Baker, Randall Armstrong, Yuqiong Pan, Dennis Leveson-Gower, Robert Negrin, Everett Meyer

**Affiliations:** 1 Division of Blood and Marrow Transplantation, Department of Medicine, Stanford University School of Medicine, Stanford, CA, United States of America; 2 Department of Medicine II, Eberhard Karls University, Tübingen, Germany; 3 Hematology and Clinical Immunology, Department of Medicine, University of Perugia, Perugia, Italy; Beth Israel Deaconess Medical Center, Harvard Medical School, UNITED STATES

## Abstract

The adoptive transfer of CD4^+^CD25^+^Foxp3^+^ regulatory T cells (Tregs) in murine models of allogeneic hematopoietic cell transplantation (HCT) has been shown to protect recipient mice from lethal acute graft-versus-host disease (GVHD) and this approach is being actively investigated in human clinical trials. Here, we examined the effects of cryopreservation on Tregs. We found that freeze and thaw of murine and human Tregs is associated with reduced expression of L-selectin (CD62L), which was previously established to be an important factor that contributes to the *in vivo* protective effects of Tregs. Frozen and thawed murine Tregs showed a reduced capacity to bind to the CD62L binding partner MADCAM1 *in vitro* as well as an impaired homing to secondary lymphoid organs *in vivo*. Upon adoptive transfer frozen and thawed Tregs failed to protect against lethal GVHD compared with fresh Tregs in a murine model of allogeneic HCT across major histocompatibility barriers. In summary, the direct administration of adoptively transferred frozen and thawed Tregs adversely affects their immunosuppressive potential which is an important factor to consider in the clinical implementation of Treg immunotherapies.

## Introduction

CD4^+^CD25^+^Foxp3^+^ regulatory T cells (Tregs) are an important T-cell population that controls immune responses and maintains immune homeostasis. Strategies to enrich donor grafts with donor-derived Tregs can protect against lethal acute graft-versus-host disease (GVHD) without compromising graft-versus-tumor (GVT) effects in murine models of allogeneic hematopoietic cell transplantation (HCT) across major histocompatibility barriers.[1] Recent clinical trials from the groups of Perugia and Minnesota that investigated adoptively transferred Tregs proved their safety and efficacy for preventing GVHD in humans.[2, 3] The potential confounding effects of freeze and thaw of Tregs have never been fully assessed.

Cryopreservation of cells is associated with a reduced viability and a reduced expression of certain surface receptors on live cells.[4–7] For example, L-selectin (CD62L) expression on CD34^+^ stem cells and Tregs was found to be decreased significantly after freezing and thawing procedures.[8–12] CD62L is an important T-cell homing receptor which binds to glycosylation-dependent cell adhesion molecule 1 (GLYCAM1) and mucosal vascular addressin cell adhesion molecule 1 (MADCAM1); expression of these proteins orchestrate T-cell trafficking and for instance, enables Tregs to enter secondary lymphoid organs.[13–15] Importantly, previous murine studies have demonstrated that adoptively transferred Tregs require CD62L expression in order to protect from lethal GVHD and autoimmunity.[16–19]

Here we investigated the impact of freezing and thawing on the CD62L expression of Tregs, their ability to home to secondary lymphoid organs and to protect from GVHD.

## Materials and Methods

### Mice

C57BL/6 and BALB/c mice were purchased from The Jackson Laboratory (Sacramento, CA). Luciferase-expressing (*luc* ^*+*^) C57BL/6 mice were created as described previously.[20] Mice were monitored daily and GVHD scores were assessed as described previously.[21] Animals with GVHD scores ≥8 were euthanized by exposure to CO_2_. Unexpected deaths did not occur. The inhalational anesthetic isoflurane was administered during bioluminescence imaging (BLI). Animal protocols were approved by the Institutional Animal Care and Use Committee of Stanford University.

### Bone marrow transplantation

BALB/c mice were conditioned with total body irradiation (2x400 cGy, 200 kV X-ray source; Kimtron), injected with 5x10^6^ T-cell depleted bone marrow (TCD-BM) cells and 5x10^5^ purified Tregs at day 0 followed by 1x10^6^ CD4^+^/CD8^+^ conventional T cells (Tcons) from C57BL/6 or *luc* ^*+*^ C57BL/6 mice at day +2. If stated, Tregs were incubated with purified Anti-CD62L (Mel14; BioLegend) for 1 hour at 4°C prior to injection.

### Freeze and thaw procedure

Freshly purified murine Tregs were resuspended in fetal bovine serum (FBS; Life Technologies) containing 10% dimethyl sulfoxide (DMSO; Sigma-Aldrich) immediately followed by freezing at -80°C in a freezing container that ensured gradual cooling of the cryotubes for at least 24 hours before transfer to a liquid nitrogen tank. Murine Tregs were stored in liquid nitrogen between 48 hours and several month depending on the experiment. For all transplantation experiments Tregs were stored for a minimum of 7 days. Mouse cells were thawed quickly in a 37°C water bath and washed in phosphate-buffered saline (PBS; Life Technologies) containing 10% FBS.

Human Tregs from granulocyte colony-stimulating factor (G-CSF)-mobilized peripheral blood apheresis products of healthy donors were frozen in 7.5% DMSO (Protide), 3% human serum albumin (HSA; Grifols), 30% Hetastarch (Hospira) in a controlled rate LN2 freezer (Custom Biogenic Systems) before cells were stored in a monitored LN2 vapor phase freezer. The mean time of human Tregs in liquid nitrogen was 33 days (range, 12–105 days). Human Tregs were thawed in a 37°C water bath and then washed with 10 volumes of Normosol (Hospira) + 2% HSA. The study of human Tregs has been approved by the Stanford University Institutional Review Board and was conducted according to the principles expressed in the Declaration of Helsinki. Healthy donors provided written consent to participate in this study which was in accordance with the Stanford University Institutional Review Board.

### Cell isolation and flow cytometry

Tcons were prepared from C57BL/6 splenocytes and lymph nodes and enriched with CD4 and CD8 MicroBeads (Miltenyi Biotec). TCD-BM cells were prepared by flushing murine tibiae and femora with PBS supplemented with 2% FCS followed by depleting T cells with CD4 and CD8 MicroBeads (Miltenyi Biotec) reaching a purity >99%. To isolate and purify Tregs, single cell suspensions from C57BL/6 spleens and lymph nodes were MACS-enriched (Miltenyi Biotec) for CD25^+^ T cells and sorted for CD4^+^CD25^bright^ T cells on a FACSAria II cell sorter (BD Biosciences). The purity of Tregs after fluorescence-activated cell sorting and after the thaw procedure was >95%. Cells were analyzed on a LSR II flow cytometer (BD Biosciences) using the following fluorochrome-labeled monoclonal antibodies purchased from BioLegend, eBioscience or BD Biosciences: CD4 (RM4-5), CD8α (53–6.7), CD25 (PC61.5), Foxp3 (FJK-16s), CD62L (Mel-14). Fixable viability dye eFluor 450 (eBioscience) was used to stain dead cells.

### In vitro MADCAM1 Binding assay

Equal numbers of live fresh and thawed *luc* ^*+*^ Tregs were incubated on plate-bound MADCAM1 for 30 min at 37°C followed by 15 min at room temperature. Wells were washed, incubated with Cell Tyter Glo (100 μl/well; Promega) and imaged with an IVIS spectrum imaging system (Xenogen). Quantitative analysis was performed based on serial dilution curves.

### In vivo bioluminescence imaging

BLI was performed as described previously (Xenogen).[22] Briefly, firefly luciferin (Biosynth) was injected intraperitoneally 10 min prior to image acquisition with an IVIS spectrum imaging system (Xenogen). Images were analyzed with Living Image Software 4.2 (Xenogen).

### Statistical analysis

Differences in animal survival (Kaplan-Meier survival curves) were analyzed with the log-rank test. All other comparisons were performed with the Student’s t test. *p*<0.05 was considered statistically significant.

## Results

### Thawing of frozen Tregs reduces CD62L expression and inhibits the binding to MADCAM1

**Fig 1:**
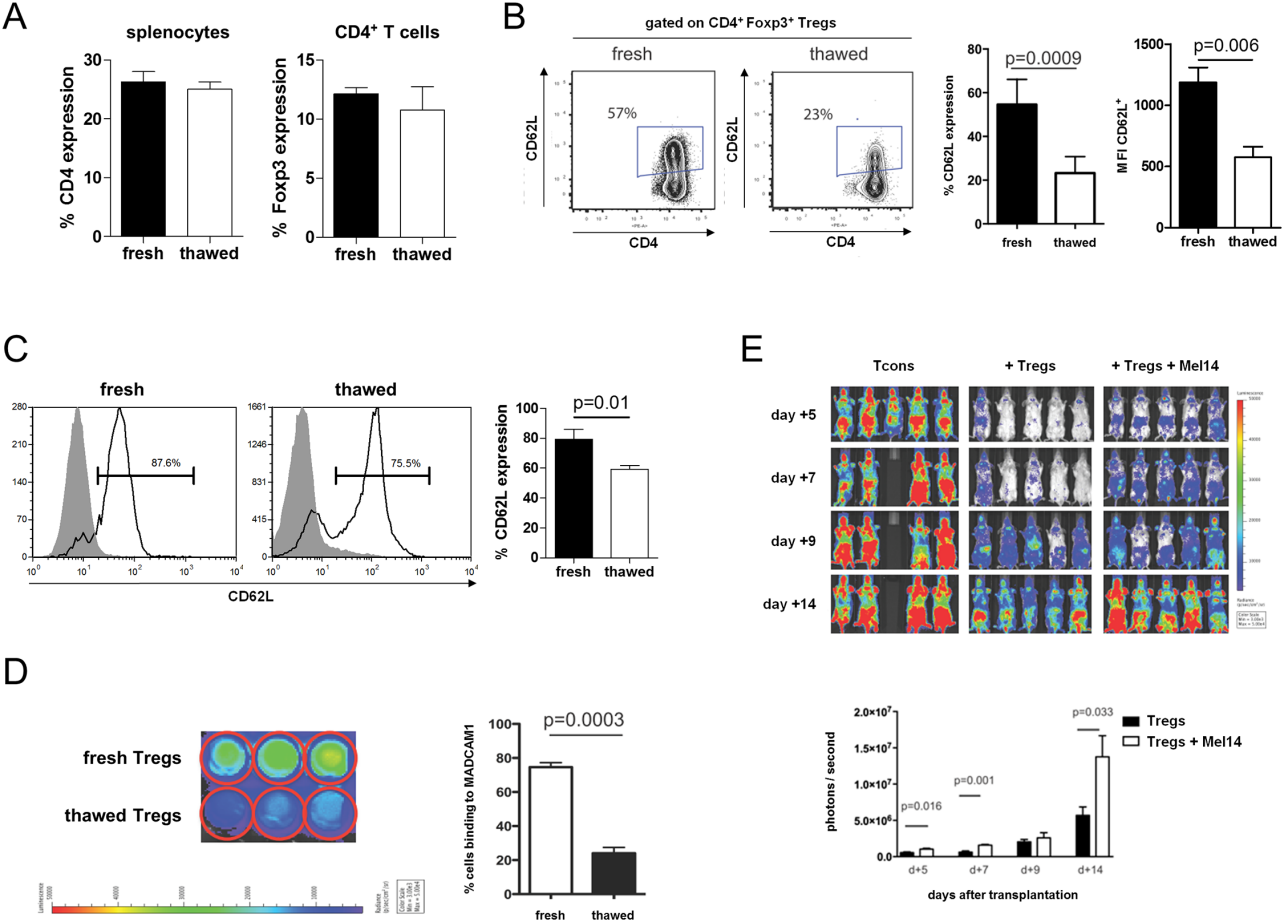
Thawing of frozen Tregs reduces CD62L expression and inhibits the binding to MADCAM1. (**A**) Relative number of CD4^+^ T cells among live splenocytes and Tregs among CD4^+^ T cells before freezing and after thawing. (**B**) Representative dot plots illustrating CD62L expression on Tregs before freezing and after thawing. Dot plots are gated on live CD4^+^Foxp3^+^ T cells. Relative number of CD62L expressing cells among Tregs. Mean fluorescence intensity (MFI) of fluorescein isothiocyanate coupled to Anti-CD62L of CD62L^+^ Tregs. Shown are 4 mice per group from one of at least two independent experiments. (**C**) Representative histograms showing CD62L expression on human CD4^+^Foxp3^+^ Tregs before and after cryopreservation. Shown are 4 single donor samples per group. (**D**) Representative bioluminescence images of isolated Tregs that adhere to plate-bound MADCAM1 *in vitro* using Cell Titer Glo. Shown is one of at least two independent experiments performed in triplicates. (**E**) Representative bioluminescence images of *luc* ^*+*^ Tcons in mice receiving Tcons alone or Tcons with fresh Tregs incubated without or with CD62L-blocking antibody (Mel14) prior to transplantation. Shown are five animals per group.

Previous reports describe a reduced CD62L expression on various cells due to cryopreservation which is a routine technique in cell processing and cellular therapy. Therefore, we measured CD62L expression by flow cytometry on murine Tregs before freezing and after thawing. Freeze and thaw did not change the relative number of CD4^+^ T cells and Tregs (Fig 1A). However, relative CD62L expression (p<0.001) and the mean fluorescence intensity of CD62L (p<0.01) on murine Tregs were both significantly decreased (Fig 1B).

Additionally, we analyzed human Tregs from G-CSF-mobilized peripheral blood apheresis products of healthy donors by flow cytometry. Thawing of these cryopreserved cells also resulted in a significantly decreased expression of CD62L (p = 0.01, Fig 1C) underlining the clinical implications of freeze and thaw procedures for Treg cellular immunotherapy in human beings.

**Fig 2:**
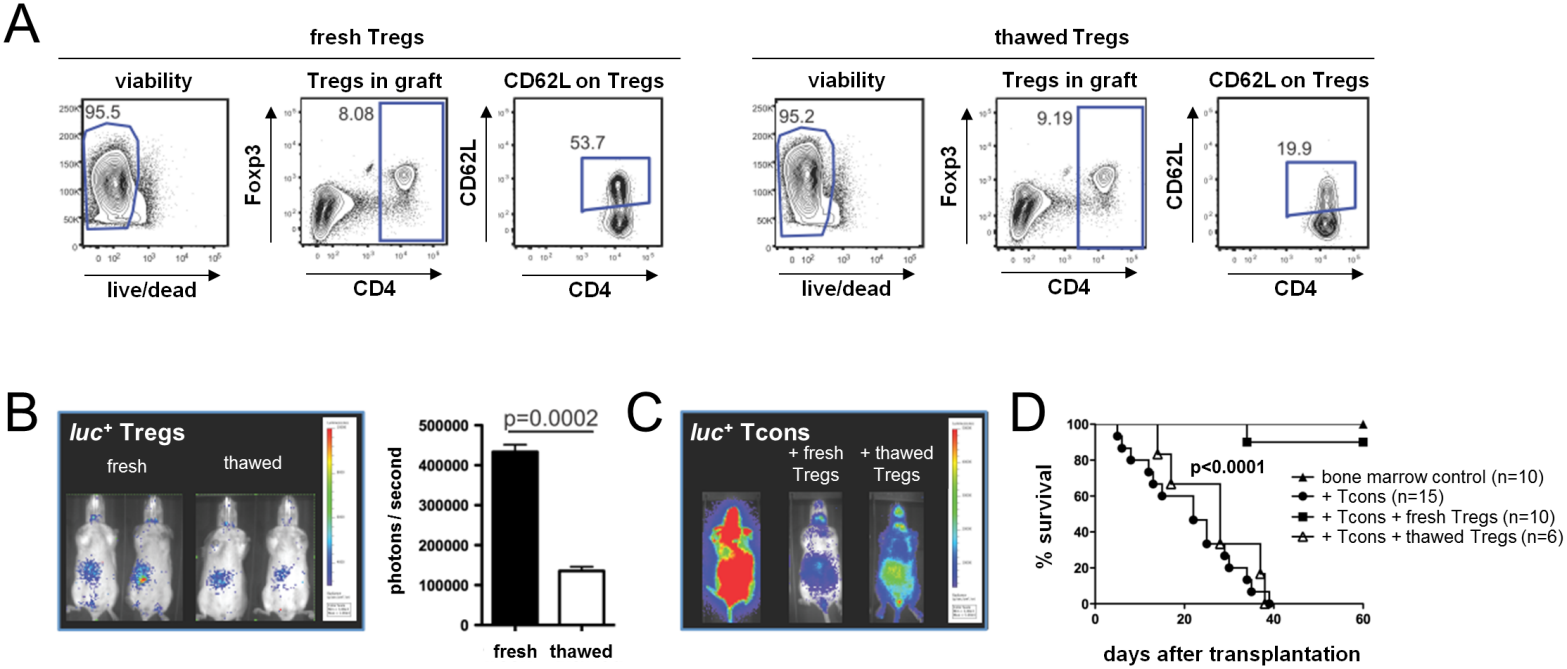
Thawed Tregs show impaired homing and fail to protect against lethal GVHD. Recipient mice received either fresh or thawed Tregs together with TCD-BM on day 0. GVHD was induced by injection of Tcons on day +2. (**A**) Representative dot plots of donor grafts before transplantation illustrating relative Treg numbers and CD62L expression on Tregs. Grafts contain equal numbers of either freshly isolated or thawed Tregs. Representative bioluminescence images from day +5 of mice transplanted with (**B**) *luc* ^*+*^ Tregs and (**C**) *luc* ^*+*^ Tcons, respectively. Shown is one of at least two independent experiments. (**D**) Kaplan-Meier survival curves pooled from two independent experiments. Number of mice per group indicated in brackets.

We wondered whether the reduced expression of CD62L on thawed Tregs would impact their ability to bind to MADCAM1 since this receptor-ligand interaction is considered critical for T-cell homing to inflamed mucosal tissues and mesenteric lymph nodes.[23] We found a significantly decreased bioluminescence signal intensity deriving from thawed *luc* ^*+*^ Tregs compared with fresh Tregs in an *in vitro* MADCAM1 binding assay (p<0.001; Fig 1D). This finding suggests an impaired *in vivo* function of cryopreserved adoptively transferred Tregs. We were able to recapitulate the requirement of CD62L for the function of Tregs through blocking the CD62L interaction with its ligands by incubating fresh Tregs with the monoclonal antibody Mel14 prior to adoptive transfer. As assessed by BLI, the expansion capacity of *luc* ^*+*^ Tcons was significantly increased in BALB/c recipient mice that received adoptively transferred Tregs pretreated with Mel14 (Fig 2E).

### Thawed Tregs fail to protect mice from lethal GVHD

Ermann et al. previously reported that adoptively transferred Tregs require expression of CD62L to protect recipient mice from GVHD.[16] The ability of Tregs to enter the priming sites of alloreactive T cells is a prerequisite for their protective function in GVHD. Therefore, we hypothesized that the loss of CD62L expression on Tregs through freeze and thaw affects their *in vivo* migration pattern after adoptive transfer as well as their capability to ameliorate GVHD.

BALB/c recipient mice were irradiated with 8 Gy followed by transplantation of TCD-BM and equal numbers of either fresh or thawed Tregs from C57BL/6 mice followed by injection of Tcons two days later. Flow cytometry of donor grafts confirmed that CD62L expression on thawed Tregs was significantly decreased (Fig 2A). Tregs that were derived from *luc* ^*+*^ C57BL/6 mice allowed us to study their trafficking pattern *in vivo*. BLI of recipient mice at day +5 revealed that the ability of thawed Tregs to enter and accumulate in secondary lymphoid organs was significantly decreased (p<0.001; Fig 2B). Consequently, the ability of cryopreserved Tregs to suppress the expansion capacity of alloreactive *luc* ^*+*^ T cells was impaired compared with fresh Tregs as measured by BLI on day +5 (Fig 2C). BALB/c mice that were treated with thawed Tregs did not show a survival benefit compared with recipient mice that received freshly purified Tregs protecting these animals from lethal GVHD (p<0.0001, Fig 2D).

## Discussion

Treg immunotherapy is an appealing approach to prevent or treat GVHD after allogeneic HCT and a variety of clinical studies have recently shown promising results in humans.[2, 3] Cellular processing techniques and logistic issues play a central role for the feasibility of this approach and they may impact the biology of Treg function.[24] In this study, we demonstrate in a murine model of allogeneic HCT across major histocompatibility barriers that freeze and thaw changes both phenotype and function of Tregs. CD62L expression was reduced after thawing of cryopreserved Tregs which resulted in an impaired ability to home to secondary lymphoid organs and to protect recipient animals from lethal GVHD as compared with freshly purified Tregs.

Previous murine studies showed that homing of adoptively transferred Tregs to secondary lymphoid organs is a prerequisite for their ability to inhibit the expansion of alloreactive T cells and that only the CD62L^+^ subpopulation of Tregs prevents lethal GVHD.[16, 18] Our findings confirm the tight connection between CD62L expression, homing to lymphoid tissues and protection from GVHD. The freeze and thaw process—a common procedure in clinical practice—is impairing the function of initially potent Tregs by altering the expression of CD62L.

Importantly, the use of a previously cryopreserved G-CSF-mobilized peripheral blood stem cell (PBSC) graft might affect the incidence of GVHD. Parody et al. reported that patients receiving frozen PBSC grafts had a higher incidence of acute GVHD mostly involving skin although Tregs were not further evaluated in this study.[25] In another study by Medd et al. the incidence of acute GVHD was comparable; however, extensive chronic GVHD at one year was more common in patients that received cryopreserved grafts.[26] Moreover, graft manipulations themselves such as mobilization of stem cells by G-CSF could potentially affect the expression of Treg surface molecules which could be a potential factor in observed differences between peripheral blood mobilized stem cells and bone marrow grafts.[27, 28]

Whether CD62L expression on Tregs could be restored after thawing is subject of ongoing investigations. In some studies, resting cells after thaw resulted in the restoration of surface molecule expression.[9, 11] However this is not always a tenable practice in clinical cellular therapy as preservatives such as DMSO can result in cell death. Moreover, resting cells and requisite washings may add a substantial risks of contamination with infectious agents.

In summary, our study highlights important caveats of a standard cell processing technique namely freeze and thaw for Treg function that need to be considered when establishing standard operating procedures for clinical protocols and organizing clinical trials investigating the preventive and therapeutic role of adoptively transferred Tregs in humans.
